# A spatial transformation-based CAN model for information integration within grid cell modules

**DOI:** 10.1007/s11571-023-10047-z

**Published:** 2024-01-02

**Authors:** Zhihui Zhang, Fengzhen Tang, Yiping Li, Xisheng Feng

**Affiliations:** 1 The State Key Laboratory of Robotics, Shenyang Institute of Automation, Chinese Academy of Sciences, No.114, Nanta Street Heping District, Shenyang, 110016 Liaoning China; 2 University of Science and Technology of China, No.96, JinZhai Road Baohe District, Hefei, 230026 Anhui China; 3 Institutes for Robotics and Intelligent Manufacturing, Chinese Academy of Sciences, No.135, Chuangxin Road Hunnan District, Shenyang, 110169 Liaoning China

**Keywords:** Grid cell, Place cell, Continuous attractors network, Path integration

## Abstract

The hippocampal-entorhinal circuit is considered to play an important role in the spatial cognition of animals. However, the mechanism of the information flow within the circuit and its contribution to the function of the grid-cell module are still topics of discussion. Prevailing theories suggest that grid cells are primarily influenced by self-motion inputs from the Medial Entorhinal Cortex, with place cells serving a secondary role by contributing to the visual calibration of grid cells. However, recent evidence suggests that both self-motion inputs and visual cues may collaboratively contribute to the formation of grid-like patterns. In this paper, we introduce a novel Continuous Attractor Network model based on a spatial transformation mechanism. This mechanism enables the integration of self-motion inputs and visual cues within grid-cell modules, synergistically driving the formation of grid-like patterns. From the perspective of individual neurons within the network, our model successfully replicates grid firing patterns. From the view of neural population activity within the network, the network can form and drive the activated bump, which describes the characteristic feature of grid-cell modules, namely, path integration. Through further exploration and experimentation, our model can exhibit significant performance in path integration. This study provides a new insight into understanding the mechanism of how the self-motion and visual inputs contribute to the neural activity within grid-cell modules. Furthermore, it provides theoretical support for achieving accurate path integration, which holds substantial implications for various applications requiring spatial navigation and mapping.

## Introduction

Spatial cognition is crucial for rodents to find food and return to their nests (Wagatsuma and Yamaguchi 2007). The concept of a cognitive map was first proposed as an abstract map to represent the environment within the brains of animals (Tolman 1948). The entorhinal-hippocampal circuit is widely thought to provide the material foundation for the cognitive map (McNaughton et al. 2006). In particular, spatial-related cells, including head direction cells, place cells, speed cells, and grid cells, have been gradually discovered in the entorhinal-hippocampal circuit. One of the most important discoveries is grid cells located in the medial entorhinal cortex (MEC) (Moser et al. 2017) due to their special firing patterns. Grid cells fire at multiple firing fields that are arranged in hexagonal structures (Rowland et al. 2016). They are widely found in many animals, including rats, mice (Fyhn et al. 2008), bats (Yartsev et al. 2011), monkeys (Killian et al. 2012), and humans (Jacobs et al. 2013; Kunz et al. 2015; Doeller et al. 2010). This shows that grid cells widely exist in the MEC and play an important role in the cognitive map (Dang et al. 2021). Since the discovery of grid cells, their function in the cognitive map has been drawing attention from researchers all the time(Zeng and Si 2021; Yan et al. 2016). One critical question about grid cells is how the hexagonal firing patterns are formed. Many models have been proposed to explain the mechanism. From the perspective of spatial dimensions, these models can be categorized into 2D models and 3D models (Wang et al. 2021b, a; Grieves et al. 2021). Given that all animals must navigate in 3D space, the exploration of 3D grid-cell models becomes crucial. However, decoding how grid cells encode 3D space remains a significant challenge, primarily due to the limited availability of biological evidence. Currently, the majority of progress in this field continues to concentrate on 2D grid-cell models (O’Keefe and Burgess 2005; Burgess et al. 2007; Hasselmo et al. 2007; Pastoll et al. 2013; Burgess 2008; Baker and Olds 2007; Fuhs and Touretzky 2006; Burak and Fiete 2009; Guanella et al. 2007; Shipston-Sharman et al. 2016; Couey et al. 2013; Kropff and Treves 2008; Samu et al. 2009; Rennó-Costa and Tort 2017; Agmon and Burak 2020). They can be divided according to information flow in the circuit between the hippocampus and MEC. The information flow within the circuit consists of three primary pathways: firstly, grid cells in the MEC serve as the primary inputs for place cells in the hippocampus; secondly, place cells act as the primary inputs for grid cells; and thirdly, there is a dynamic interplay between grid cells and place cells, both of which play equally essential roles in the circuit. In the following, we delve into a comprehensive discussion of the information flow within the circuit and explore related models in detail.

In the early stages, it is widely believed that grid cells serve as the primary contributors of inputs for place cells. Upon the initial discovery of grid cells, Hafting et al. (2005) noted that grid cells exhibited sustained stability in the absence of visual input, suggesting that the formation of grid fields might result from the integration of idiothetic self-motion cues. Specifically, the sequential flow of sensory information from the entorhinal cortex to the hippocampus has been observed in previous anatomical investigations, including the seminal work by Felleman and Van Essen in monkeys (Felleman and Van Essen 1991). Consequently, the hierarchical organization of visual regions positions the hippocampus at the apex while receiving inputs from the entorhinal cortex (Zhong and Wang 2021). Therefore, the prevailing hypothesis in the field suggests that grid cells may serve as a neural representation of the spatial environment, relying on path integration. Despite the spatial tuning exhibited by both grid cells and place cells, there are notable distinctions in their spatial firing characteristics. Grid cells demonstrate a distinctive spatial oscillatory pattern, whereas place cells typically exhibit single-peaked firing fields. Various studies have provided evidence that the integration of multiple grid cell activities can give rise to place-cell firing fields through the summation of their inputs (McNaughton et al. 2006). The summation process results in the disappearance of grid fields at most positions while reinforcing specific positions, ultimately contributing to the sparse nature of place-cell firing activity. Based on the forward postulate discussed above, some grid-cell models are proposed to simulate the process in the circuit of the hippocampus and the medial entorhinal cortex. The models can be simply divided into two types, namely, Oscillatory-interference (OI) models (O’Keefe and Burgess 2005; Burgess et al. 2007; Hasselmo et al. 2007; Pastoll et al. 2013; Burgess 2008; Baker and Olds 2007) and continuous attractor network (CAN) models (Fuhs and Touretzky 2006; Burak and Fiete 2009; Guanella et al. 2007; Shipston-Sharman et al. 2016; Couey et al. 2013). They utilize the speed and head direction information from speed cells and head direction cells in the MEC and generate the hexagonal firing patterns to provide inputs for place cells.

However, recent evidence demonstrates the place cells from the hippocampus may play an important role in the formation of hexagonal patterns. Evidence from studies indicates that during development, there is a precedence of place cell-like activity before the emergence of grid cells (Langston et al. 2010). Place-cell activity in rats can be observed as early as 16 days of age, during the puppy stage. In contrast, the activity of grid cells is typically first recorded at around 20 days of age in rats. The observation suggests that place cells may not necessarily require input from grid cells for their formation or functionality. More experimental evidence shows that the grid cells need inputs from the hippocampus. As an illustration, studies have shown that inhibiting the septum can disrupt the firing patterns of grid cells while leaving the activity of place cells unaffected (Koenig et al. 2011). Additionally, global remapping of place cells in the hippocampus can occur when the inputs from the medial entorhinal cortex (MEC) are severed (Schlesiger et al. 2018). Compelling evidence supporting the essential role of place-cell activity in grid-cell pattern formation comes from studies where hippocampal inactivation leads to the loss of the grid pattern in the medial entorhinal cortex (MEC) neurons (Bonnevie et al. 2013). This finding highlights the dependence of grid-cell pattern formation on the presence and functionality of place-cell activity within the hippocampus. According to the aforementioned discussion and supporting evidence, the consensus indicates a predominant directionality of information flow from place cells to grid cells. The single-cell plasticity models are designed to align with empirical evidence. Compared with other grid-cell models, the single-cell plasticity models place more emphasis on external sensory cues from the hippocampus and learning processes (D’Albis 2018; D’Albis and Kempter 2017).

Recent findings indicate that the relationship between grid cells and place cells is potentially more intricate than previously conceived, thereby challenging the adequacy of simplistic interpretations of their interconnection (Morris and Derdikman 2023; Yan et al. 2016). For instance, the inhibition of MEC inputs to the hippocampus does not influence the firing fields of place cells. Conversely, the depolarization of stellate grid cells can provoke a tendency for place cells to remap (Kanter et al. 2022). Furthermore, there is evidence suggesting that enlargement in the grid-cell scale correlates with a corresponding expansion in the scale of place fields (Mallory and Giocomo 2018). In an attempt to elucidate the relationship between the hippocampus and the MEC, several loop models have been proposed (Samu et al. 2009; Rennó-Costa and Tort 2017; Agmon and Burak 2020). These models propose that the reciprocal exchange of information between grid cells and place cells is of equal significance for their respective functions. At the heart of these models lies the provision of redundancy of grid cells and place cells for spatial representation. To a certain extent, the loop models facilitate understanding of the information flow in the circuitry between the hippocampus and the MEC. However, the interaction between place cells and grid cells remains an open question.

As mentioned previously, the information flow between the hippocampus and MEC has been the subject of extensive research. Nevertheless, the transfer and processing of information within this circuit remain enigmatic. The prevailing theory suggests that grid cells receive self-motion inputs from MEC, while place cells offer a visual calibration for grid cells (Bush et al. 2015). However, recent evidence manifests that place cells may not only serve functions beyond simply providing visual calibration for grid cells (Schlesiger et al. 2018; Kanter et al. 2022; Mallory and Giocomo 2018), which may collaboratively contribute to the formation of grid-like patterns. In this paper, we propose a novel CAN model based on a spatial transformation mechanism to give place cells more roles for the formation of grid patterns. This mechanism transforms external cues and self-motion inputs into grid-cell cognitive space via place cells. By this, the self-motion inputs and visual cues from place cells can be synergistically integrated to form grid-like patterns and drive the network activity. Considering individual neurons within the network, our model adeptly replicates grid firing patterns. At the level of neural population activity, the network is capable of forming and propelling the activated bump, exhibiting the important feature of grid-cell modules, path integration. Further experiments demonstrate that our model exhibits significant path-integration performance. In addition, our model acquires a more natural arrangement of neurons within the network compared to the classical grid-cell CAN model (Guanella et al. 2007) due to the spatial transformation mechanism. In conclusion, this study provides a new insight into understanding the mechanisms of how self-motion and visual inputs contribute to neural activity within grid-cell modules. Moreover, it provides theoretical support for achieving accurate path integration, which facilitates the development of brain-inspired spatial navigation and mapping.

For clarity, we have summarized the main contributions of our research as follows:

- We construct a CAN model for grid modules based on spatial transformation to synergistically process the self-motion and visual inputs. Further results demonstrate that the individual grid-cell neurons within the network can successfully reproduce the firing patterns. Notably, our model achieves a more natural arrangement of neurons within the network, in contrast to the manual organization, as seen in other current CAN models.
- The path integration using our model is performed by processing self-motion and visual cues. Our model exhibits significant path-integration performance. Moreover, although grid-cell spacing and network sizes do have an impact on the accuracy of path integration, our model effectively constrains error within a finite range.
- This study provides a new insight into understanding the mechanisms by which self-motion and visual inputs contribute to neural activity within grid-cell modules. Additionally, it offers theoretical support for achieving precise path integration, which contributes to the development of brain-inspired spatial navigation.

## Results

**Fig. 1:**
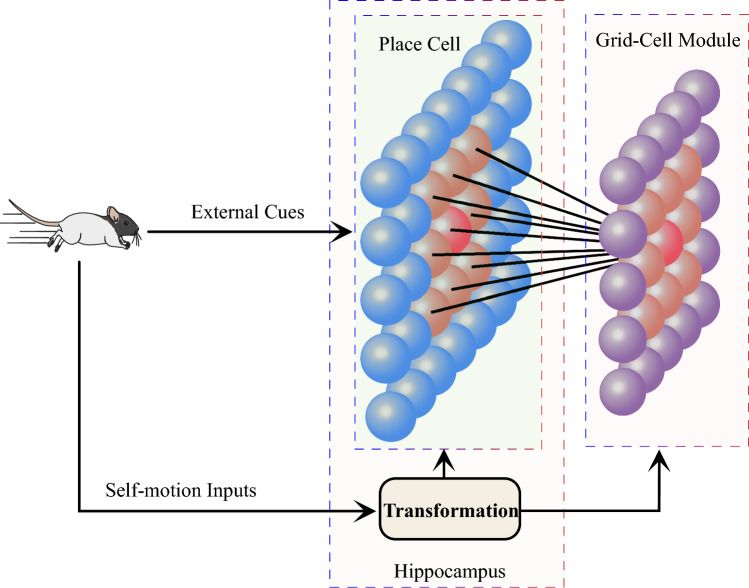
The framework of information flows from the hippocampus to grid-cell modules, which synergistically integrates external cues and self-motion inputs. The self-motion inputs are transformed into place-cell cognitive space and grid-cell cognitive space. The processed information is then used to drive the place-cell model and grid-cell modules. During the movement, the place cells code the external cues from MEC. When the rat revisits the same place in the actual world, the place cell coding the same position is activated. The excitatory from place cells are transformed into grid-cell modules to eliminate the accumulated error. In addition, the external cues also shape the place-cell cognitive space gradually, indirectly influencing the grid-cell patterns by affecting the transformation module

The entorhinal-hippocampal circuit, a fundamental part of the brain’s cognitive system, has been a subject of immense interest in neuroscience. This circuitry, located within the medial temporal lobe, plays a crucial role in spatial navigation. It involves two main regions: the hippocampus and the entorhinal cortex, which are interconnected in a complex manner that facilitates a diverse array of cognitive functions. Empirical evidence demonstrates that there are reciprocal anatomical connections between grid cells and place cells. However, the relationship between grid cells and place cells is not fully understood, but it is generally believed that they work together to support navigation and spatial memory. Place cells within the circuit are generally thought to provide visual calibration for grid cells. However, recent research suggests that place cells may play a more critical or determining role in influencing the activity of grid cells (Bonnevie et al. 2013). Here, we propose a framework to process information flow from the hippocampus to grid-cell modules. The whole framework is described in Fig. 1. As Fig. 1 depicted, the self-motion inputs and external cues all are initially transformed into the hippocampus. After the procession of the hippocampus, the information is then processed by grid-cell modules in MEC to generate the firing activity.

We first introduce the CAN model for grid-cell modules. The information flow within grid-cell modules for self-motion inputs and external cues is then described.

### The structure of the proposed CAN model

**Fig. 2:**
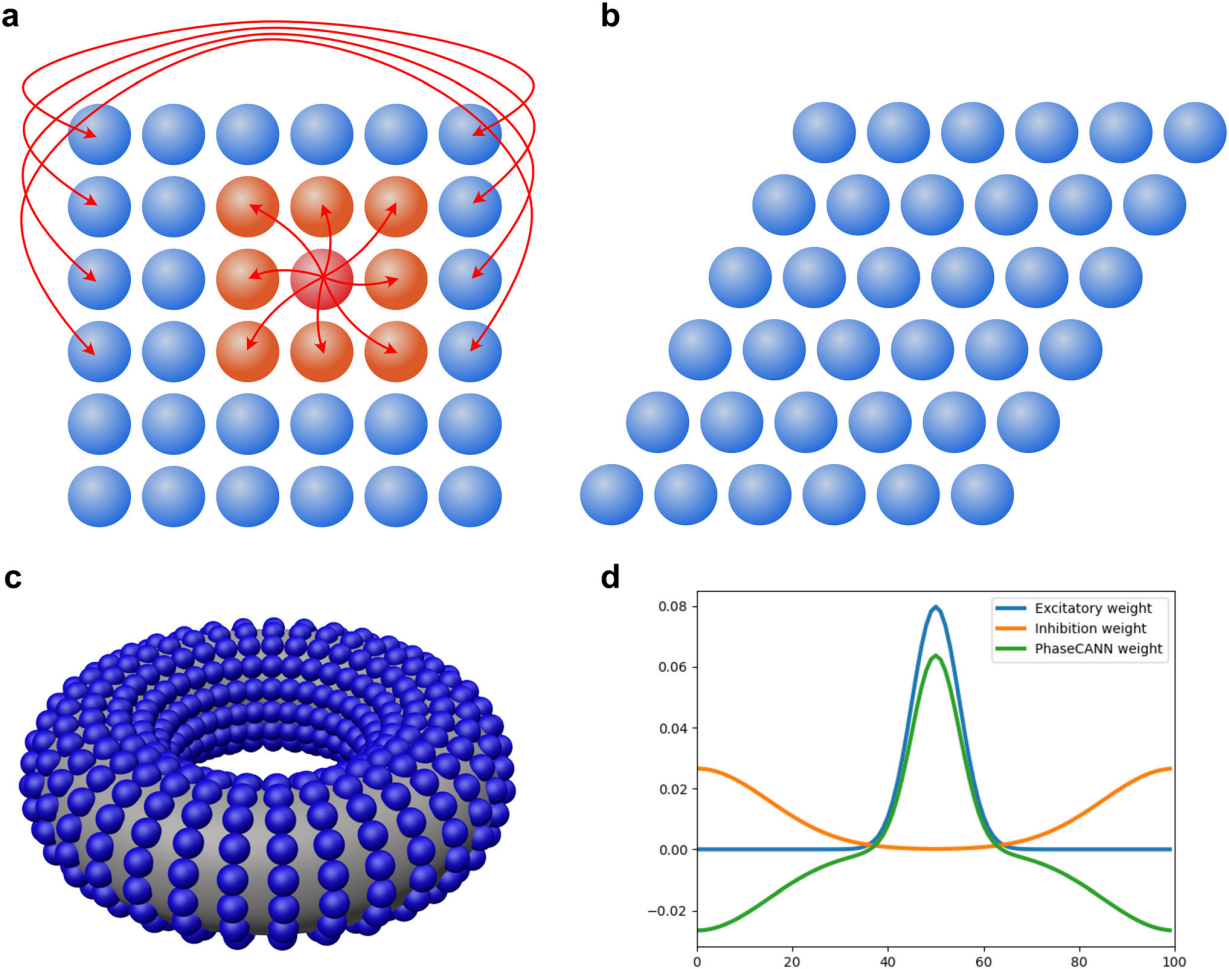
The structure of the proposed model. **a** The arrangement of grid-cell neurons of the proposed CAN model. The neurons in a singular grid-cell module are arranged on a sheet according to their phases. They are mutually connected by weight profile according to the distance between them on the sheet. The distance between neurons needs to consider the periodic boundary condition. **b** The arrangement of grid-cell neurons in the CAN model in the Guanella et al. (2007). **c** The networks with the periodic boundary condition can form a toroidal manifold. In the toroidal manifold, the neurons are mutually connected according to their weights. **d** The weight profile of the proposed model. The weight profile of the proposed model consists of two parts. One part is the excitatory weight to activate the local neurons. The other part is the inhibition part, and it inhibits distant neurons. The two parts are used to compute the weight profile of the proposed model by Eq. (12)

In contrast to the arrangement shown in Fig. 2b, our model does not require a 60-degree angle configuration as suggested by Guanella et al. (2007). As illustrated in Fig. 2a, in our model, the neurons within the same grid-cell module naturally form a planar arrangement. Within this sheet, the neural population is defined as follows:

1 $$\begin{aligned} M = N_x \cdot N_y \end{aligned}$$

where *M* represents the total number of neurons, $N_x$ is the number of neurons in the horizontal direction, and $N_y$ is the number of neurons in the vertical direction. In our model, the relationship of $N_x$ and $N_y$ are defined by:

2 $$\begin{aligned} N_x = N_y = N \end{aligned}$$

As widely acknowledged, grid cells possess three distinctive attributes: spacing, phase, and orientation. To facilitate the description of grid cells in the proposed model, the *i*-th grid cell in the network is denoted as follows:

3 $$\begin{aligned} \varvec{\epsilon _i} = [s_i,o_i,\varvec{\vartheta _i}] \end{aligned}$$

where $s_i$ is spacing of the grid-cell $\varvec{\epsilon _i}$, $o_i$ is the orientation of the grid cell $\varvec{\epsilon _i}$, $\varvec{\vartheta _i}$ is the phase of the grid cell $\varvec{\epsilon _i}$, and $\varvec{\vartheta _i} = [\vartheta _i^1,\vartheta _i^2]$ contains the phases of the two directions. For the grid cells in the same module denoted as ${\mathcal {M}}$, they share common spacing and orientation. So the grid cells in a module can be further represented as follows:

4 $$\begin{aligned} \varvec{\epsilon _i} = [s,o,\varvec{\vartheta _i}], i\in {\mathcal {M}} \end{aligned}$$

A grid cell $\varvec{\epsilon _i}$ within a module can be mapped onto a 2D sheet based on its phases. As a result, each grid cell $\varvec{\epsilon _i}$ is associated with a coordinate pair (*x*, *y*) in the 2D sheet, which we label as $\varvec{\epsilon _{xy}}$. Their relationship can be described as follows:

5 $$\begin{aligned} {} \varvec{\epsilon _i} = \varvec{\epsilon _{xy}} \quad \text {if} \quad i = N \cdot x + y \end{aligned}$$

Different from the CAN model in (Guanella et al. 2007), all neurons in our model are arranged as a matrix instead of a repetitive rectangular structure. According to the grid-cell phases, the index (*x*, *y*) of a neuron $\varvec{\epsilon _{xy}}$ in the matrix can be described as follows:

$$\begin{aligned} {\left\{ \begin{array}{ll} x = \frac{\vartheta _i^1}{\Delta \vartheta ^1} \qquad \qquad (6)\\ y = \frac{\vartheta _i^2}{\Delta \vartheta ^2} \qquad \qquad (7) \end{array}\right. } \end{aligned}$$

where $\Delta \vartheta ^1$ and $\Delta \vartheta ^2$ are the phase gaps of neighboring neurons along two directions, $\vartheta _i^1$ and $\vartheta _i^2$ are the phases of $\varvec{\epsilon _i}$. As Fig. 2c depicted, the neurons in our model are connected recurrently and have the periodic boundary condition. Thus the maximum phases of the neurons is equal to the spacing of the grid module. Then $\Delta \vartheta$ can be further calculated as follows:

8 $$\begin{aligned} \Delta \vartheta = \frac{s}{N} \end{aligned}$$

where *s* is the grid-cell spacing, and *N* represents the size of the CAN along either the x-axis or y-axis.

### The connection of the proposed model

As mentioned above, the grid cells in a module are arranged in a sheet. They are interconnected and have a periodic boundary condition (see Fig. 2c). According to (Burak and Fiete 2009), the firing dynamics of these grid cells that have the same spacing and orientation can be described by:

9 $$\begin{aligned} \tau \frac{d r_{i}}{d t}+r_{i}=f\left[ \sum _{j} w_{i j} r_{j}\right] \end{aligned}$$

where $\tau$ is the time constant of neuron response, $r_i$ is spike rate of the neuron, and *f* is a non-linear function given as follows:

$$\begin{aligned} f(x)= {\left\{ \begin{array}{ll} =x, x>0 \qquad \qquad (10)\\ =0, otherwise \qquad (11) \end{array}\right. } \end{aligned}$$

The $w_{ij}$ in Eq. (9) is the connection weight from neurons $\varvec{\epsilon _j}$ to neuron $\varvec{\epsilon _i}$. It comprises two parts. The first part is used to excite the neighboring neurons of the neuron $\varvec{\epsilon _j}$. The second part is utilized to inhibit the neighboring neurons of the neuron $\varvec{\epsilon _j}$. Here, two Gaussian functions are used to generate the weight profile and their domain satisfies the periodic boundary condition (see Fig. 2d). Consequently, $w_{ij}$ can be described as follows:

12 $$\begin{aligned} w_{ij}=\alpha e^{-\rho d_{ij}^2} - \beta e^{-\gamma (d_{ij}-D)^2} \end{aligned}$$

where $\alpha ,\beta$ control the extent of the Gaussian function, $\rho ,\gamma$ adjust the scope of the Gaussian function, *D* is the max distance between any two neurons in the network, $d_{ij}$ is the distance between $\varvec{\epsilon _j}$ and $\varvec{\epsilon _i}$, and it can be computed as follows:

13 $$\begin{aligned} d_{ij}&= ||\varvec{\epsilon _i} - \varvec{\epsilon _j}||_2 \end{aligned}$$

14 $$\begin{aligned}&=\sqrt{(x_i-x_j)^2+(y_i-y_j)^2} \end{aligned}$$

### The information flow of external cues

Place cells have been considered to provide inputs for grid cells (Li et al. 2020). The grid-like patterns observed in the medial entorhinal cortex (MEC) of rats were found to be lost after the hippocampus was temporarily inactivated, as recorded through neural firings. The receptive fields of grid cells in this condition become tuned to the direction of the rat’s head (Bonnevie et al. 2013).

According to our mechanism, the information from the MEC is first transferred into the hippocampus. This information is then transformed into cognitive space. For a landmark in the physical world, we regarded it as a visual cue to provide an input for place cells. According to our theory (Zhang et al. 2023), the way that the place cells receive external cues is to transform them into place-cell cognitive space. The frames for the physical world and place-cell cognitive space are different in our model. So the landmark $P_i^w$ in the physical world needs to be transformed, which includes rotation and translation. A landmark $P^w_i$ is first transformed into place-cell cognitive space by:

15 $$\begin{aligned} \varvec{P^p_i} = \varvec{R_{wp}}\cdot \varvec{P^w_i} + \varvec{\varpi _i} \end{aligned}$$

where $\varvec{R_{wp}}$ and $\varvec{\varpi }$ are the rotation matrix and translation vector, respectively. They are determined by the difference between the world frame and the place-cell frame. The difference can be further represented by the rotation angle $\phi$ as follows:

16 $$\begin{aligned} \varvec{R_{wp}} = \begin{bmatrix} cos\phi &{} -sin\phi \\ sin\phi &{} cos\phi \end{bmatrix}^{-1} \end{aligned}$$

In this way, the landmark can be utilized to inject energy into the place-cell CAN model (McNaughton et al. 2006). The injected energy can then change the activities of the place cells. After the process of network dynamics, the state of the place cell network is gradually stabilized because of the reciprocal connections among place cells in the sheet.

The neuron that has the maximum firing rate is identified, representing the position $\varvec{P^p_\text {max}}$. It will be transformed and described in grid-cell firing space by:

17 $$\begin{aligned} \varvec{P^g_i}&= \varvec{R_{pg}}\cdot \varvec{P^p_\text {max}} \end{aligned}$$

where $\varvec{P^g_i}$ is the corresponding neuron position in the CAN and $\varvec{R_{pg}}$ is the transformation matrix. It can be depicted as follows:

18 $$\begin{aligned} \varvec{R_{pg}} = \begin{bmatrix} s\cdot cos(o_i)&{} s\cdot cos(o_i+\pi /3)\\ s\cdot sin(o_i)&{} s\cdot sin(o_i+\pi /3) \end{bmatrix}^{-1} \end{aligned}$$

where *s* and *o* are the spacing and orientation of the grid-cell module, respectively. The injected energy at position $\varvec{P^g_i}$ generally can be calculated as follows (Fuhs and Touretzky 2006):

19 $$\begin{aligned} {\tilde{r}}_i = \sum _{k=1}^{M} { {T}}_{ik} {{F}}(\varvec{P^p_k}) \end{aligned}$$

where *M* is the neural population of the place-cell network, ${{F}}(\cdot )$ is the function to acquire the firing rate of $\varvec{P^p_k}$ in the place-cell network, and ${{T}}_{ik}$ is the weight profile. This weight profile is initially defined using a 2D Gaussian kernel function and can then be updated by Hebbian rule (Chakraverty et al. 2019). But in this paper, to avoid performing image matching and simplify our experiments, the energy injected at position $\varvec{P^g_i}$ is set equal to the firing rate of $\varvec{P^p_\text {max}}$, i.e.,

20 $$\begin{aligned} {{\tilde{r}}}_i = { {F}}(\varvec{P^p_\text {max}}) \end{aligned}$$

where ${ {F}}(\cdot )$ is the function to acquire the firing rate of $\varvec{P^p_k}$ in the place-cell network.

Through the mechanism mentioned above, the proposed model can acquire excitation from external cues. The firing rate of single grid cell in the Eq. (9) then can be further described as follows:

21 $$\begin{aligned} \tau \frac{d r_i}{d t}+r_i=f\left[ \sum _j w_{i j} r_j+{\tilde{r}}_i\right] \end{aligned}$$

where ${{\tilde{r}}}_i$ is the feed-forward input to neuron *i* from external cues. After receiving the external excitation, the proposed model needs to process the excitatory update. A two-dimensional discrete Gaussian distribution is used to generate the excitatory weight matrix, $w_{ij}$, which is depicted in Eq. (12). Each neuron then uses it to project activity to all other neurons in the proposed model. Finally, the proposed model is normalized to constrain the sum of activation in the whole network. Before normalization, the firing rate of each neuron must be constrained within a range by the Heaviside function. The normalization process can be described as follows:

22 $$\begin{aligned} r_i:= \frac{r_i}{\sum _{j=1}^{M} r_j} \end{aligned}$$

where *M* is the neural population of the proposed model, as described in Eq. (1). Furthermore,

23 $$\begin{aligned} {}&r_i = r_{xy}\quad \text {if}\quad i = N \cdot x + y \end{aligned}$$

where *N* represents the size of the CAN model along the x-axis, *x* and *y* are the indices in the network sheet. By this, we can determine the position of neuron *i* within the network sheet.

**Fig. 3:**
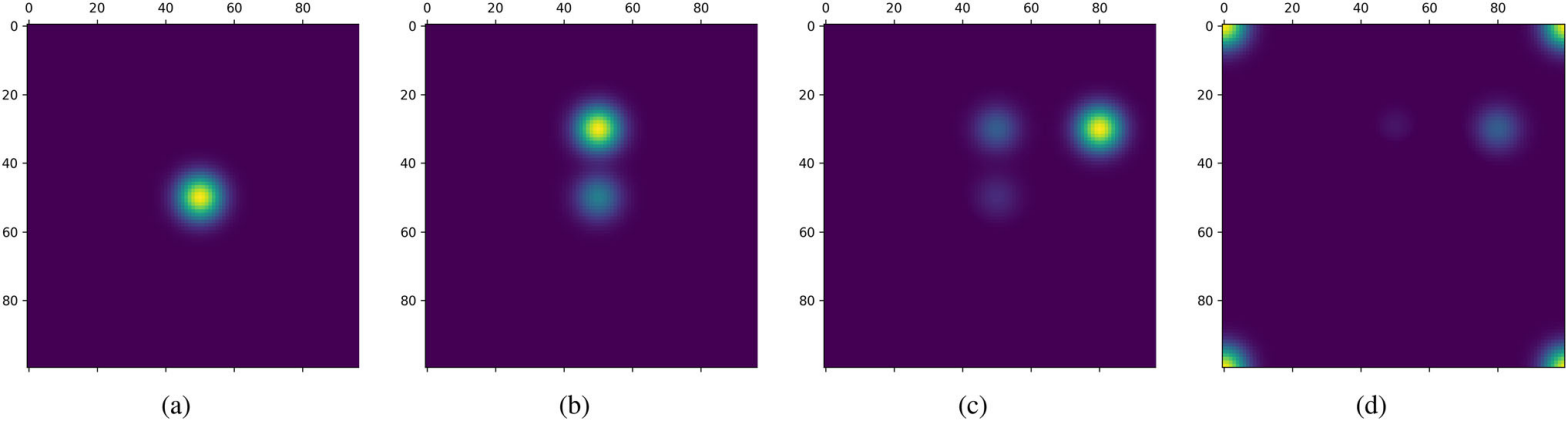
Activity and stabilization. The yellow color and blue color represent the high and low excited regions separately. **a** Firstly, the energy is injected into the network at the position (50, 50). The energy is spread by synaptic weight profile and the activity bump is formed in the sheet with position (50, 50) as its center. **b** Then, the energy is injected into the network at a new position (50, 30). The internal dynamics drive the network to stabilize. The new position (50, 30) is mostly activated forming a dominated activity bump and the previous position becomes much less activated, becoming a dim bump. **c** Similar to **b**, the energy is injected into the network at another new position (80, 30). Similar phenomena are observed. **d** Slightly different from (**b**), the energy is injected into the network at the edge (100, 0). The activity bump appears in the four corners of the network, due to the periodic boundary condition

To assess the network’s capacity to receive excitation from external cues, we conducted experiments with $N=100$, spacing $s=80~\textrm{cm}$, the orientation $o=0$ and other parameters outlined in Table 1. The experimental outcomes are illustrated in Fig. 3.

**Table 1:** The parameters of the proposed model

Parameters	$\alpha$	$\beta$	$\rho$	$\gamma$
Value	1.0	1.0	0.01	0.003

As Fig. 3 depicted, the proposed model can receive the stimulation and remain stable by dynamic adjustment. We continuously injected energy in different positions (From Fig. 3a–c). This injection of energy gives rise to the formation of activity bumps, facilitated by excitation connections among proximal neurons and inhibition connections among distant neurons. Importantly, the energy injected in a subsequent position inhibits the energy injected in the preceding position. Consequently, the activity bump shifts in response to changes in the position of injected energy. Notably, remnants of prior activity bumps can still be observed, as demonstrated in Fig. 3b, c. From Fig. 3c, we can see that effects from the activity bump in Fig. 3a almost disappear. The earlier activity bump disappears faster than the later-formed activity bump. This observation indicates that our model possesses appropriately weighted connections between neurons to facilitate excitation and inhibition. Notably, When the bump position is on the edge of the network sheet, the bump will split into two parts, appearing on the corner of the network sheet, as shown in Fig. 3d. This behavior is attributed to the periodic boundary condition.

### The information flow of self-motion inputs

CAN-based grid cell models offer distinct advantages in path integration compared to other grid cell models. Path integration is achieved through the dynamic adjustment of the network’s activity bump, driven by self-motion inputs from speed cells (Kropff et al. 2015) and head direction cells (Zhang 1996; Taube et al. 1990).

However, the precise manner in which self-motion inputs contribute to grid cells remains an ongoing inquiry. In the context of CAN-based grid-cell models, there are typically two primary methods for shifting the activity bump within the network (Milford and Wyeth 2008). One way is to project the existing neural activity bump to the expected future location and leave the competitive dynamics of the CAN model to form the activity bump in the future location. It takes time and effort to stabilize the internal network dynamics. Thus, the performance of path integration in this way is highly affected by the sensory update rates and robot velocity. The other way is to directly move the existing neural activity bump by rotation or translation, without re-stabilizing internal network dynamics. Thus sensory update rates and robot velocity have little impact on its performance, which makes it obtain more precise trajectories and eliminate the need for parameter adjustment (Milford and Wyeth 2008). In this paper, our primary emphasis is on the network activity that is raised by external inputs. Therefore, we have opted for the latter method, which accurately simulates the actual biological process. This choice helps us avoid introducing new sources of noise that could potentially impact the results of our models.

However, different to Milford and Wyeth (2008), which can directly use the translation in the world frame, our method needs to encode the translation in the grid-cell space by the proposed model. The firing rate of the expected future location is computed through convolutional kernel operations.

After the rat moves a distance per unit of time, the offsets can be calculated as follows:

24 $$\begin{aligned} \begin{aligned} \Delta x^w_i&= \int _{t_{i}}^{t_{i+1}}|\varvec{v}(t)| \cdot cos\alpha (t) \ \text {d}t \\ \Delta y^w_i&= \int _{t_{i}}^{t_{i+1}}|\varvec{v}(t)| \cdot sin\alpha (t) \ \text {d}t \end{aligned} \end{aligned}$$

where $\Delta x^w_i$ and $\Delta y^w_i$ are the translation of two directions in the world frame, $\varvec{v}(t)$ and $\alpha (t)$ are the translation velocity and head direction, respectively. Then the translation in the world frame can be encoded by the proposed model via:

25 $$\begin{aligned} \left[ \Delta x^g_i,\Delta y^g_i\right] ^T&= \varvec{R_{pg}}\cdot \varvec{R_{wp}} \left[ \Delta x^w_i,\Delta y^w_i\right] ^T \end{aligned}$$

where $\Delta x^g_i$ and $\Delta y^g_i$ are the offsets in the proposed model, $\varvec{R_{wp}}$ and $\varvec{R_{pg}}$ respectively derive from Eqs. (16) and (18).

For a neuron $\epsilon _{xy}$, its firing rate $r_{xy}$ can be updated by the path integration as follows:

26 $$\begin{aligned} r_{xy} = \sum _{a=\delta x_o }^{\delta x_o +1}\sum _{b=\delta y_o}^{\delta y_o +1} \eta _{ab} r_{(x+a)(y+b)} \end{aligned}$$

where $\eta _{ab}$ is a $2\times 2$ convolution kernel used to compute the firing rate of $r_{xy}$, *a*, *b* are the integral indices. Every item of $\eta _{ab}$ can be acquired as follows:

27 $$\begin{aligned} \eta _{ab} = \frac{\sqrt{\left( a-\Delta x_i^g\right) ^2+\left( b-\Delta y_i^g\right) ^2}}{\sum _{a=\delta x_o }^{\delta x_o +1}\sum _{b=\delta y_o}^{\delta y_o +1}\sqrt{\left( a-\Delta x_i^g\right) ^2+\left( b-\Delta y_i^g\right) ^2}} \end{aligned}$$

where $\delta x_o,\delta y_o$ are the rounded-down integer offsets in the *x* and *y* directions. They can be calculated as follows:

28 $$\begin{aligned} \begin{bmatrix} \delta x_o\\ \delta y_o \end{bmatrix} = \begin{bmatrix} \left\lfloor \Delta x_i^g \right\rfloor \\ \left\lfloor \Delta y_i^g \right\rfloor \end{bmatrix} \end{aligned}$$

where $\lfloor \cdot \rfloor$ represents the floor operation.

The data obtained by the neurobiological experiment (Hafting et al. 2005) was used here. During the movement of the rat, its velocity and angle were recorded. These data were used to drive the proposed network. The grid-cell module is configured in the same way as our previous experiments. In addition, the world frame and the frame of place cell firing space are set up identically. In other words, the rotation matrix $\varvec{R_{wp}}$ is the identity matrix and the translation vector $\varvec{\varpi }$ is the zero vector.

**Fig. 4:**
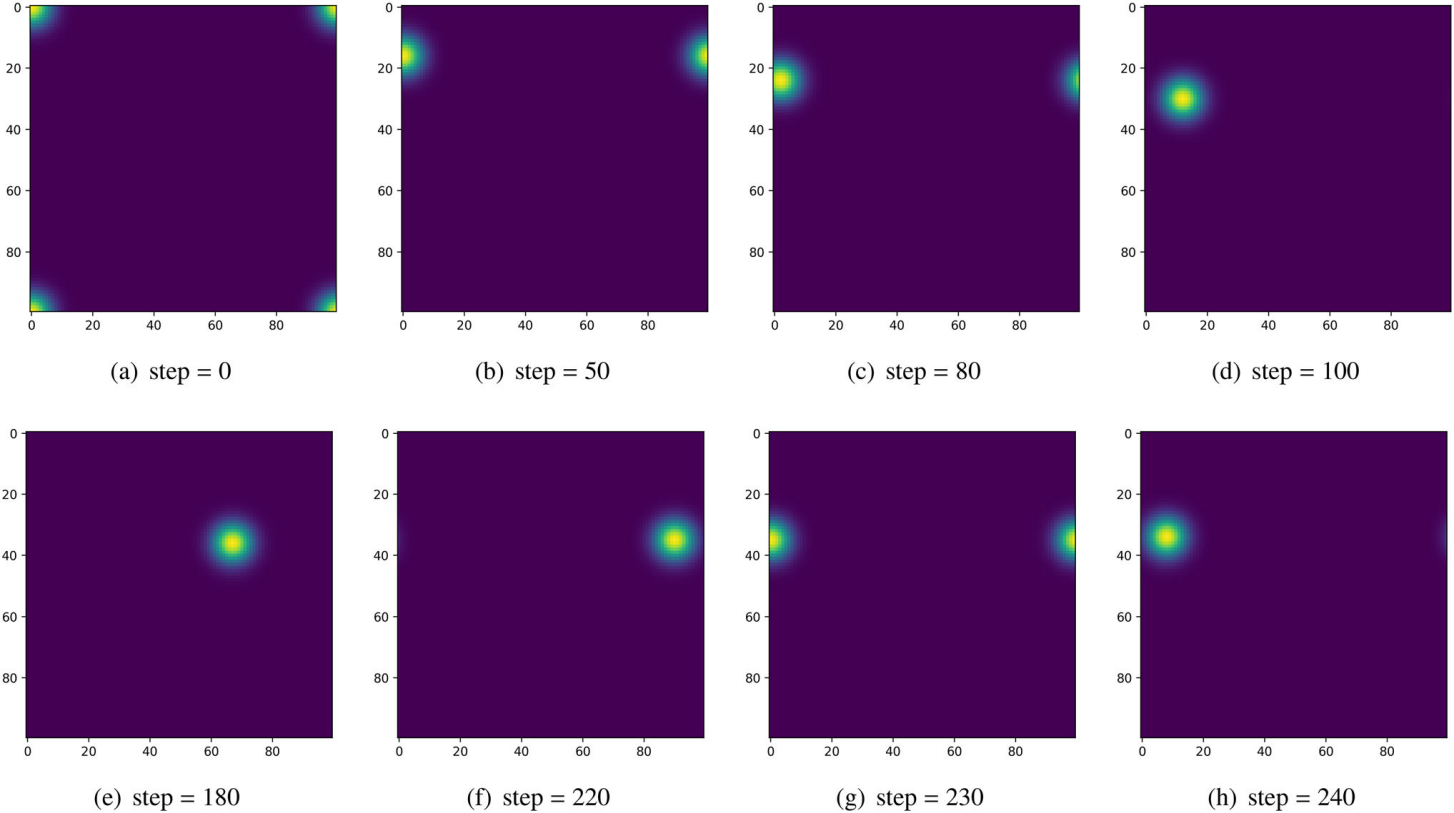
The snapshots of network status during the path integration. In the figures, different colors represent various firing rates of the neurons. The firing rates gradually decline from yellow to blue. In the beginning, as (**a**) shows, the origin point is the (0, 0) and so the corresponding neuron in the (0, 0) is activated. Along with the movement of the rat in the actual environment, the excitatory bump begins to move. In addition, the snapshots are chosen per several steps herein to better show the process

At each time step, the offsets are computed using Eq. (24), and the firing rate of each neuron in the proposed model is updated using Eqs. (25)–(28). Snapshots of the network’s status during this process are presented in Fig. 4. The activity bump can be seen moving continuously across the network sheet with the movement of the rat in the actual environment. As we can see in the figure, when the bump arrived at one edge of the network sheet, it reappeared on the opposite edge. This phenomenon comes from the periodic boundary condition, which derives from the manifold structure of cognitive space. These results demonstrate that the proposed model can maintain stability and perform path integration, even at arbitrary locations within the network.

**Fig. 5:**
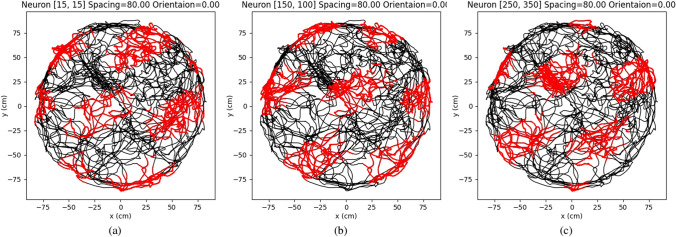
The firing patterns of the single grid cell in the different positions of the proposed model. The black lines in the three figures are the paths that are generated in the virtual environment. The red points represent the firing position of the single neuron. **a** The firing pattern of the neuron in the position (15, 15) of the network. **b** The firing pattern of the neuron in the position (150, 100) of the network. **c** The firing pattern of the neuron in the position (250, 350) of the network

To investigate the activity of a single grid cell in the module, we execute the aforementioned process in our model, employing parameters as detailed in Table 1, with $N=400$, $s=80~\textrm{cm}$, and $o=0$. Three neurons in our model, i.e. (15,15), (150,100), and (250, 350) were extracted for detailed inspection. Their firing activities during path integration are given in Fig. 5. As we can see all three neurons exhibit hexagonal symmetry firing patterns consistent with the known properties of grid cells and previous experimental results (Hafting et al. 2005).

**Fig. 6:**
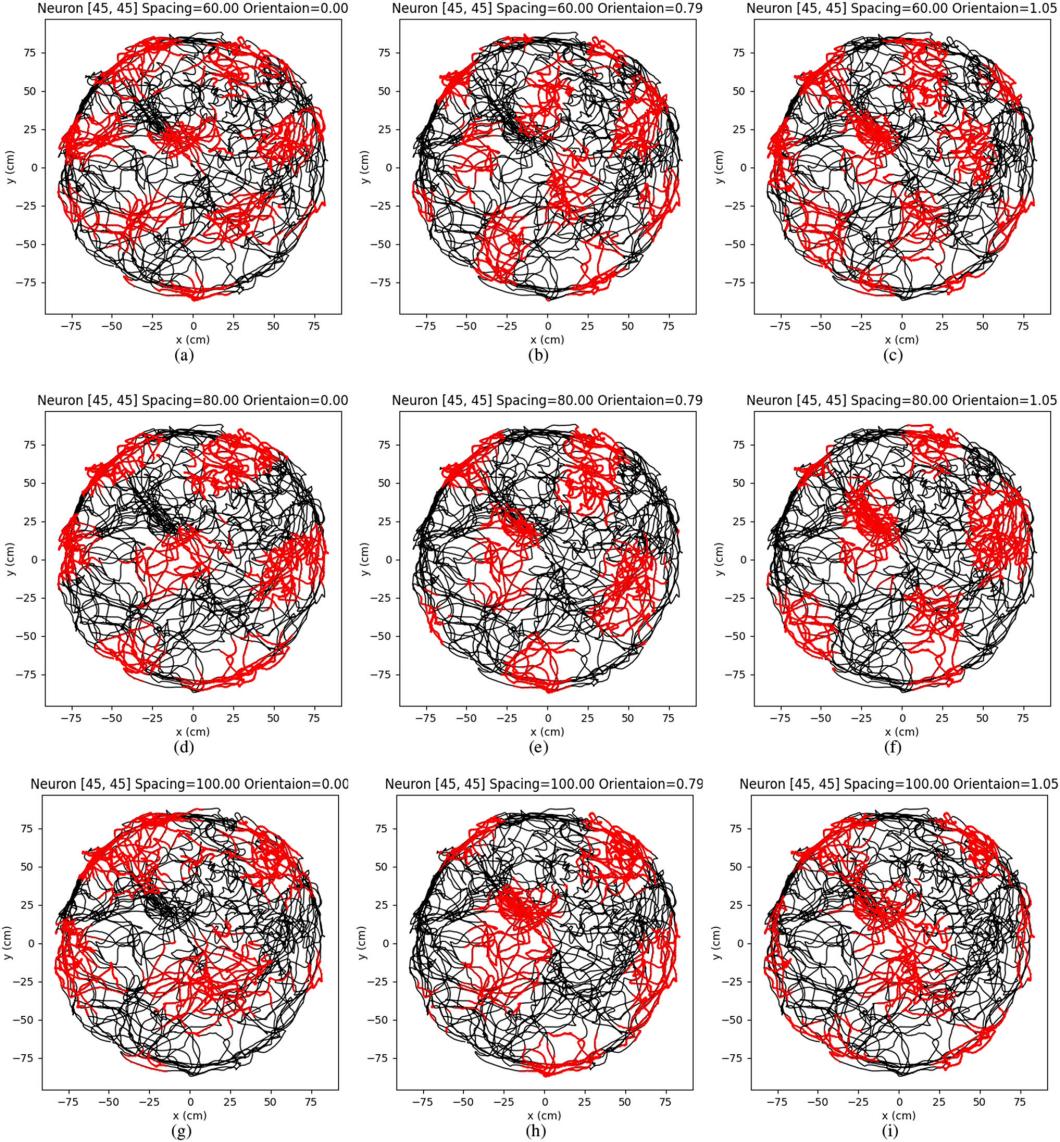
The firing patterns of the single grid cell in the different modules but in the same position of the network. The firing patterns in different orientations for the grid cell in the same position of the network are illustrated from left to right. With the increasing orientations of the grid-cell module, the grid patterns incrementally rotate. The firing patterns with different spacing of the grid-cell modules are compared from top to bottom. The scales of single grid-cell firing patterns incrementally raised with the increasing spacing of grid-cell modules

To further elucidate the activities of individual neurons across various grid cell modules, we constructed nine grid cell modules using the proposed model, systematically varying the spacings and orientations. The network size was kept as 400 in these experiments while other parameters were set as the same listed in Table 1. Subsequently, we visualized the firing activities of neurons located at identical positions within the nine CANs that were driven by the previously mentioned path, as depicted in Fig. 6.

Comparing the figures from left to right, the firing patterns are rotated with increasing orientation of the grid cell module. In addition, the change of interval is consistent with the increasing spacing of the grid cell module. This demonstrates our proposed model can stimulate different properties of grid cells. In previous research (Bush et al. 2015; Edvardsen et al. 2020), perfect gird patterns are used to perform accurate path integration. However, the actual grid-like patterns recorded by biological experiments (Hafting et al. 2005) usually are irregular and twisted, which is consistent with our results (see Figs. 5 and 6). Biological experiments also show that the grid-like patterns can be distorted by border cells (Krupic et al. 2015). Therefore the model for single grid cell may not be suitable for accurate path integration. Based on our concept, path integration should be performed using a grid-cell module rather than a single grid cell.

**Fig. 7:**
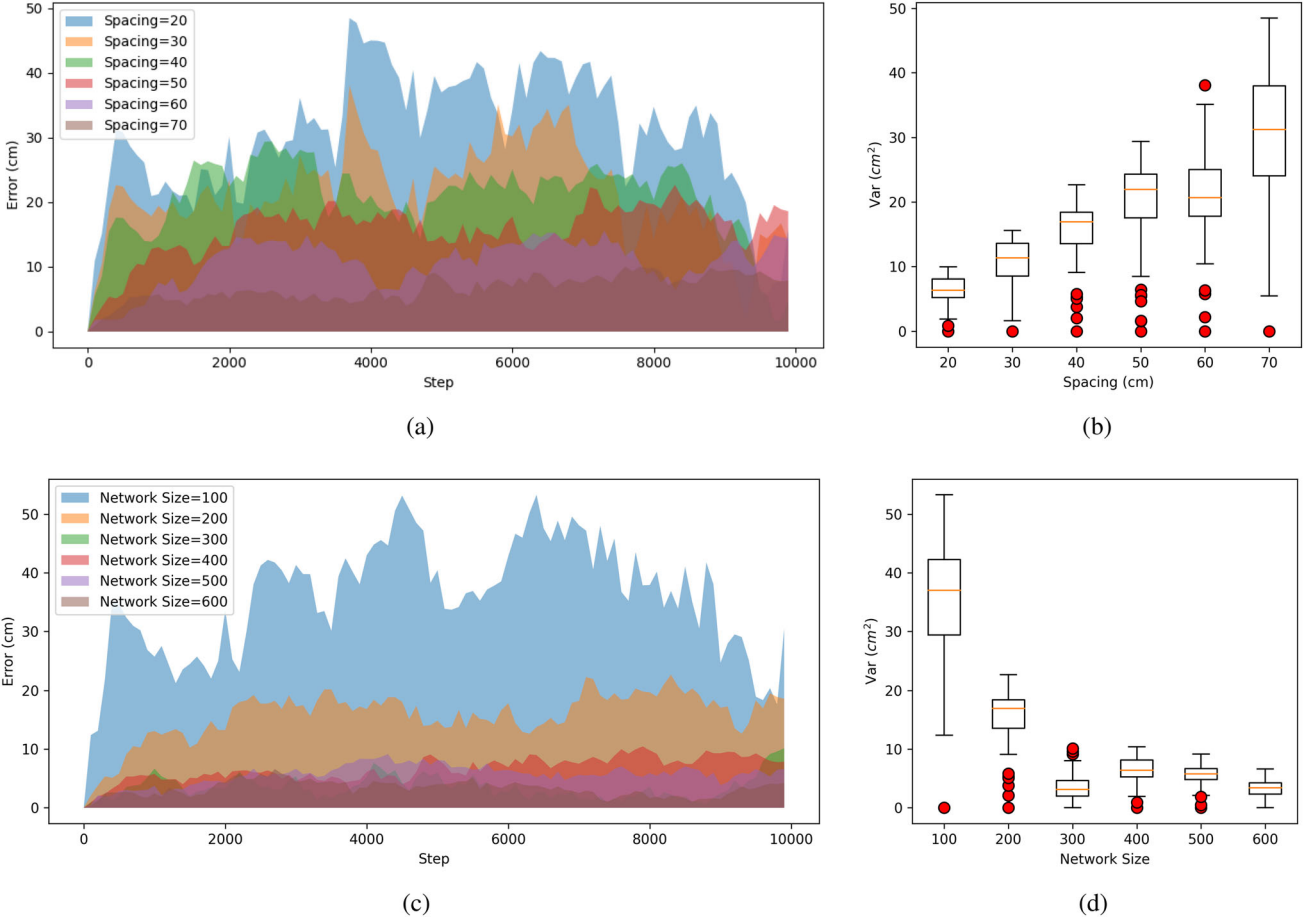
The performance of path integration using the proposed model with iterations. The x-axis is the moving steps. **a** The errors of path integration using different spacing but the same size of the network, i.e., 100. **b** The variance for different spacing but the same network size. **c** The error of path integration using different network sizes but the same spacing of grid cell, i.e., $80~\textrm{cm}$. **d** The variance for various network sizes but the same spacing

To verify the path integration ability of our model, we designed several experiments. We compared the performance of different CANs with varying spacing and network size. The errors of path integration were recorded per 100 steps and calculated by:

29 $$\begin{aligned} e = \Delta \vartheta \cdot g(P,P') \end{aligned}$$

where $\Delta \vartheta$ is the distance of adjacent neurons in the network mentioned in Eq. (8), $g(\cdot )$ is a function to compute the Euclidean distance, *P* and $P'$ separately are the expected position and reached position after the path integration. The *P* can be calculated by projecting true location into grid-cell cognitive space using Eqs. (15), (16) and (17). The results of the experiment are shown in Fig. 7.

Figures 7a, b depict the errors and variance in path integration observed during experiments that assessed the performance of varying spacings. These experiments were conducted with the proposed model’s network size consistently set to 100. As evidenced by Fig. 7a, b, the spacing of grid cells significantly influences the error and variance in path integration. Notably, larger spacings tend to exacerbate both error and uncertainty in path integration.

To examine the effects of different network sizes on path integration, we kept the grid-cell spacing constant at 80 and analyzed the resultant performance. Figures 7c, d demonstrate that smaller network sizes can notably influence the error and variance of path integration. However, for network sizes exceeding 300, the impact on error and variance becomes negligible, as illustrated in Fig. 7d.

In conclusion, regardless of variations in grid cell spacing or network size, our model consistently keeps the error smaller than $50~\textrm{cm}$, as evidenced by Fig. 7a, c. This suggests that the error in path integration doesn’t escalate indefinitely. More specifically, according to Fig. 7, the error in path integration is directly proportional to the grid cell spacing and inversely proportional to the network size.

So we can describe the relationship between the variance and the parameters of the model as follows:

30 $$\begin{aligned} e \varpropto \frac{N}{s} = \frac{1}{\Delta \vartheta } \end{aligned}$$

where *s* represents the spacing of grid cells, *N* is the size of the network along the x-axis or y-axis, $\Delta \vartheta$ is the ratio of *s* and *N* marked as network resolution ratio of CAN, as depicted in Eq. (8). The above relationship is inferred according to the results depicted in Fig. 7, which suggests the error of path integration is affected by the network resolution ratio $\varrho$. Our findings suggest that the spacing and network sizes used in our model have an impact on error. Nevertheless, their impact is limited, and our model is still able to constrain the error within a certain range. In other words, our model can maintain a certain level of accuracy performance regardless of its parameters, indicating its robustness and accuracy in navigating tasks.

### Feedback by place cells

Typically, the error in path integration accumulates over time. In robotic navigation tasks, visual cues are leveraged to correct this error. Similarly, for rodents, several studies suggest that place cells serve this corrective function (Focus on spatial cognition 2017; Moser et al. 2017; Crivelli-Decker 2023; Wang and Wang 2021). In our model, the role of place cells is simulated by projecting actual-world landmarks into the place-cell network, as illustrated in Fig. 1. When the animal returns to a previously visited location, the place cell corresponding to its landmarks becomes activated. This excitation within the place-cell network is then translated and mapped to the firing space of grid cells. The inputs from the place-cell network prompt adjustments within the grid-cell module. After stabilizing the grid-cell module, corrections to the accumulating path integration error are applied. In this manner, our proposed grid-cell module effectively addresses and eliminates accumulated path-integration error.

**Fig. 8:**
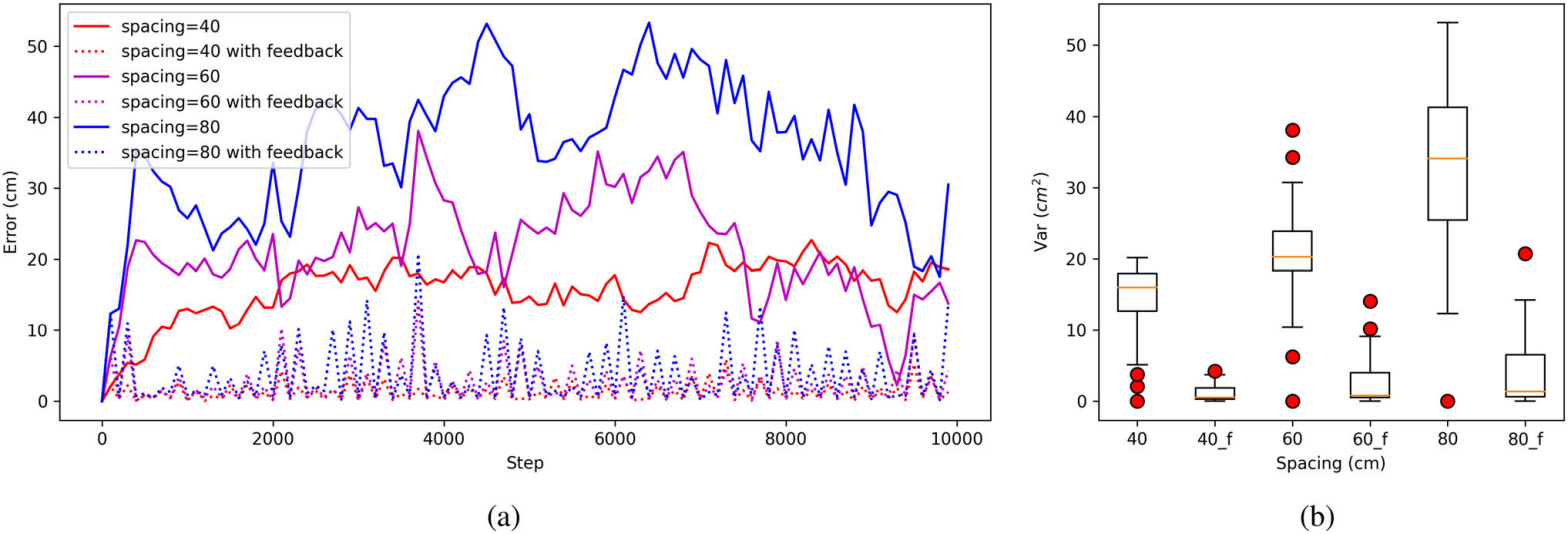
The performance of path integration with and without feedback. **a** The error comparison of path integration with and without feedback. The solid lines represent the error curves without feedback, while the dashed lines represent the error curves with feedback. **b** The variance of path integration with and without feedback. The 40_f, 60_f and 80_f in the x-axis denote the models have the feedback and their spacing are 40$~\textrm{cm}$, 60$~\textrm{cm}$ and 80$~\textrm{cm}$

For the sake of experimental simplicity and to eliminate the effects of scene recognition, external cues were selected every 200 steps and directly incorporated into the grid-cell modules. To emphasize the role of feedback in our model, we monitored the path integration error every 100 steps and juxtaposed the performance of models with and without feedback, as illustrated in Fig. 8. We also adjusted the spacing of grid-cell modules to investigate feedback’s influence under varied conditions. Results revealed that models with feedback consistently outperformed those without, exhibiting reduced errors and enhanced stability across all module spacings (refer to Fig. 8). Notably, tighter spacing between grid-cell modules yields a steadier error trajectory, denoting enhanced stability in performance (as seen in Fig. 8b). This underscores our model’s capability to counteract accumulating error through feedback mechanisms.

## Discussion

The rodent animals have outstanding navigation ability(Ball et al. 2013). They can travel a long way to forage and return to their dens precisely. This demonstrates they have the remarkable ability of path integration. However, the latent mechanism of animals’ navigation is still an open question. The cognitive map is believed to exist in the brain of animals (Tolman 1948). It can provide a spatial representation of the physical world to guide the animals. The identification of place cells provides evidence that the entorhinal-hippocampal circuitry is involved in the formation of a cognitive map (O’Keefe and Conway 1978). After that, many spatial cells are found in these areas including head direction cells, speed cells, grid cells and so on. Specially, The hexagonal firing patterns exhibited by grid cells have gathered significant attention. It is hypothesized that the regular firing patterns observed in the medial entorhinal cortex (MEC) serve as an efficient way to represent an individual’s location in large-scale space (Bush et al. 2015).

Currently, the interaction between the hippocampus and MEC is still in disputation. Prevailing views think that the place cells in the hippocampus provide a visual calibration for grid cells and grid cells mainly receive the self-motion inputs from the MEC. However, the biological evidence demonstrates that the inactivation of the hippocampus leads to the disappearance of grid cells. Therefore, some researchers think the main inputs for grid cells may come from the external cues via place cells (Kropff and Treves 2008; Si and Treves 2013; D’Albis and Kempter 2017). The grid-cell models based on this theory place a stronger emphasis on the learning process and exhibit more biological plausibility. However, recent evidence suggests that both self-motion inputs and visual cues may collaboratively contribute to the formation of grid-like patterns. In our previous work (Zhang et al. 2023), a mechanism for interaction between place cells and single grid cell has been proposed. It can replicate grid patterns and is consistent with current research (Carpenter et al. 2015). However, it has not taken grid cells within a module into consideration. In this paper, a novel grid-cell CAN model is proposed for grid-cell modules to process the information flow based on the proposed mechanism. The neurons in the proposed model are naturally arranged. We show the status of the proposed model from two levels. One is to illustrate the activities of the grid-cell modules. It shows our model can be driven and remain stable after receiving self-motion inputs and external cues. The other is to show the activities of single grid cell in the grid-cell module. It can exhibit grid firing fields when driving the grid-cell modules. We then use our model to perform the path integration and compare the results in different grid-cell properties and sizes of the proposed model. The results point out that our model can acquire good accuracy regardless of the network parameters. Furthermore, the results also manifest that our model can acquire outstanding performance even in the absence of external cues and on a large scale.

The consensus is that grid cells provide a path integration input for place cells (McNaughton et al. 2006). So the grid cells are regarded as a key component for spatial representation in the entorhinal-hippocampal circuitry. The key hypothesis for the CAN model is that path integration can be performed by moving the bump in the network on the sheet(Fuhs and Touretzky 2006). Many mechanisms are designed to shift the bump around a ring (Zhang 1996) or over a sheet (Burak and Fiete 2009). Compared with other models, our model drives the attractor bump by transforming the self-motion information into the cognitive space of grid cells. To simplify the process, the speed and head direction are calculated to acquire the translation at a small time scale. Then the translation is transformed into the cognitive space of grid cells. Through this method, path integration is obtained in our model.

However, the error in the path integration will accumulate over time. Then, the correction of accumulated error by sensory cues is a crucial challenge in path integration. Recent evidence has illustrated that the grid cells need to receive the inputs from place cells (Chen et al. 2016). So it is generally believed that the place cells play a crucial role in correcting the accumulated errors for the grid cells. In our model, the external cues are encoded by place cells. Then the representation for external cues in place cells is transformed into grid-cell modules. When the animal revisits the same location in the physical world, the place cells are activated due to the same visual cues. This stimulation in place cells are injected into grid-cell modules by Eqs. (17)–(19), as shown in Fig. 1. The accumulated errors of path integration in grid-cell modules can be corrected after the dynamic adjustment of the networks, as shown in Fig. 8. Our experimental results indicate that the accuracy and stability of path integration in our model are highly affected by the spacing and network size. However, the error of path integration in our grid cell model was confined to a certain range (see Fig.  8). Our model even can maintain low errors in a long voyage without feedback from external cues (see Fig.  7). Therefore our model partly explains why rodent animals have so a prominent ability for navigation.

## Conclusion

In this paper, we introduce a novel Continuous Attractor Network (CAN) model based on a spatial transformation mechanism. This mechanism facilitates the integration of self-motion inputs and visual cues within grid-cell modules, collectively driving the emergence of grid-like patterns. From the perspective of individual neurons within the network, our model successfully replicates grid firing patterns. When examining neural population activity within the network, it becomes evident that the network can create and sustain the activated bump-a defining characteristic of grid-cell modules, specifically, path integration. Through further exploration and experimentation, our model demonstrates significant proficiency in path integration. This study offers a new perspective on understanding the mechanisms through which self-motion and visual inputs contribute to neural activity within grid-cell modules. Moreover, it provides theoretical support to achieve robust navigation and establish a framework for bio-inspired robotic navigation systems to manage the transmission and processing of information.

In this paper, to eliminate the influence of scene recognition for evaluating the performance of the proposed model, the external cues are projected into grid-cell modules through place cells according to a certain frequency. In our future work, scene recognition (Xu et al. 2022) will be considered and added to our model. Then, the external cues can inject energy into the grid-cell module when previous encountered scene is recognized. By this, we hope the proposed model can outperform the existing bio-inspired navigation system (Ball et al. 2013).
